# Dorsolateral prefrontal circuit effective connectivity mediates the relationship between white matter structure and PASAT‐3 performance in multiple sclerosis

**DOI:** 10.1002/hbm.25239

**Published:** 2020-10-19

**Authors:** Dewen Meng, Thomas Welton, Afaf Elsarraj, Paul S. Morgan, Roshan das Nair, Cris S. Constantinescu, Nikos Evangelou, Dorothee P. Auer, Rob A. Dineen

**Affiliations:** ^1^ Radiological Sciences, Division of Clinical Neuroscience, School of Medicine University of Nottingham Nottingham UK; ^2^ Sir Peter Mansfield Imaging Centre, School of Medicine University of Nottingham Nottingham UK; ^3^ NIHR Nottingham Biomedical Research Centre, Queen's Medical Centre University of Nottingham Nottingham UK; ^4^ National Neuroscience Institute Tan Tock Seng Hospital Singapore Singapore; ^5^ Medical Physics and Clinical Engineering Nottingham University Hospitals NHS Trust Nottingham UK; ^6^ Institute of Mental Health University of Nottingham Nottingham UK; ^7^ Division of Psychiatry & Applied Psychology, School of Medicine University of Nottingham Nottingham UK; ^8^ Clinical Neurology, Division of Clinical Neuroscience, School of Medicine University of Nottingham Nottingham UK

**Keywords:** dorsolateral prefrontal circuit, effective connectivity, mediation effect, multiple sclerosis

## Abstract

Three decades ago a series of parallel circuits were described involving the frontal cortex and deep grey matter structures, with putative roles in control of motor and oculomotor function, cognition, behaviour and emotion. The circuit comprising the dorsolateral prefrontal cortex, caudate, globus pallidus and thalamus has a putative role in regulating executive functions. The aim of this study is to investigate effective connectivity (EC) of the dorsolateral‐prefrontal circuit and its association with PASAT‐3 performance in people with multiple sclerosis(MS). We use Granger causality analysis of resting‐state functional MRI from 52 people with MS and 36 healthy people to infer that reduced EC in the afferent limb of the dorsolateral prefrontal circuit occurs in the people with MS with cognitive dysfunction (left: *p* = .006; right: *p* = .029), with bilateral EC reductions in this circuit resulting in more severe cognitive dysfunction than unilateral reductions alone (*p* = .002). We show that reduced EC in the afferent limb of the dorsolateral prefrontal circuit mediates the relationship between cognitive performance and macrostrucutral and microstructural alterations of white matter tracts in components of the circuit. Specificity is shown by the absence of any relationship between cognition and EC in the analogous and anatomically proximal motor circuit. We demonstrate good stability of the EC measures in people with MS over an interval averaging 8‐months. Key positive and negative results are replicated in an independent cohort of people with MS. Our findings identify the dorsolateral prefrontal circuit as a potential target for therapeutic strategies aimed at improving cognition in people with MS.

## Graphical abstract

Multimodal neuroimaging studies allow testing of biologically plausible models of multiple sclerosis‐related cognitive dysfunction. We used multimodal MRI to demonstrate reduced effective connectivity in the dorsolateral prefrontal circuit correlates with cognitive performance in people with multiple sclerosis and mediates the relationship between structural alterations in the circuit and cognition.

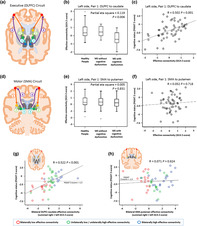

**Abbreviations**
BOLD: blood oxygen level dependent
BraNDy‐MS: imaging markers of brain network disruption in multiple sclerosis
CIS: clinically isolated syndrome
CSF: cerebrospinal fluid
DLPFC: dorsolateral prefrontal cortex
FC: functional connectivity
FDR: false discovery rate
fMRI: functional MRI
GCA: granger causality analysis
GLM: general linear model
HC: healthy control
HCP: human connectome project
LGI: local gyrification index
PASAT: paced auditory serial addition test
PCA: principal component analysis
RD: radial diffusivity
SMA: supplementary motor area
VeSPA‐MS: enous stasis and permeability assessment in multiple sclerosis
WMT: white matter tract

## 1 INTRODUCTION

Cognitive dysfunction affects ~40–70% of people with multiple sclerosis (MS), and adversely impacts employment, daily living, social interaction and potential to benefit from rehabilitation (Achiron et al., 2013; Chiaravalloti & DeLuca, 2008; Grzegorski & Losy, 2017; Hamalainen & Rosti‐Otajarvi, 2016). Across the various clinical courses of MS, deficits in processing speed, working memory and sustained attention are found (Drew, Starkey, & Isler, 2009; Manca, Sharrack, Paling, Wilkinson, & Venneri, 2018). These aspects of cognitive dysfunction are critical in many aspects of daily life. However, despite its importance, the underlying mechanisms of cognitive dysfunction in MS are not well‐understood.

A series of parallel neural circuits involving the frontal cortex and deep grey matter nuclei have been described (Alexander, 1994; Alexander, Crutcher, & DeLong, 1991; Bonelli & Cummings, 2007). These circuits have a variety of putative functional roles but have a common core architecture of frontal cortex → striatum → globus pallidus → thalamus → frontal cortex. The dorsolateral prefrontal circuit is believed to have a role in regulating executive functions (Cummings, 1993; Tekin & Cummings, 2002) and is well‐studied in the context of cognitive function in the healthy brain and other contexts such as substance use disorder (Ma et al., 2018). Focal lesions involving structures participating in this circuit, such as the DLPFC, caudate and thalamus, are widely recognised to have detrimental consequences for cognition (Caplan et al., 1990; Schmahmann, 2003; Stuss & Benson, 1984), and researchers have proposed that effective disruption to the circuit from the focal lesion can be considered as a form of ‘disconnection’ syndrome (Schmahmann & Pandya, 2008). Structural and functional disconnection has been proposed as a mechanism of cognitive dysfunction in MS (Dineen et al., 2009; Rocca et al., 2015). While studies have shown regional damage in the dorsolateral prefrontal circuit in MS patients with cognitive dysfunction, including atrophy of the dorsolateral prefrontal cortex (DLPFC), caudate or thalamus (Batista et al., 2012; Dineen, Bradshaw, Constantinescu, & Auer, 2012; Houtchens et al., 2007; Nocentini et al., 2014), previous studies have not examined the structural, functional and cognitive relationships in this circuit as a whole.

Unlike functional connectivity, which examines the statistical correlation of blood oxygen level dependent (BOLD) signal in different brain regions, effective connectivity (EC) allows inference on how one region influences another region in the brain. Granger causality analysis (GCA) provides a hypothesis‐driven method allowing estimation of the influence of one brain region on activity in another brain region through top‐down mechanisms (Friston, 1994; Friston Moran, & Seth, 2013; Iwabuchi et al., 2017; Tomasi, Wang, Wang, & Volkow, 2014). The presence of GCA from one region X to another region Y implies that the neuronal activity in region X precedes and predicts the neuronal activity in region Y. Thus, EC measured by Granger causality analysis (GCA) provides a directional hypothesis‐driven inferential method allowing estimation of the connectivity within the dorsolateral prefrontal circuit through top‐down mechanisms. While GCA of fMRI data has previously been controversial, it is a powerful tool when applied properly and carefully (Seth, Barrett, & Barnett, 2015). For example, GCA‐EC has previously been used to show breakdown of salience‐execution loop in schizophrenia, identifying that reorganisation of salience network could be a treatment target in schizophrenia (Palaniyappan, Simmonite, White, Liddle, & Liddle, 2013). GCA‐EC is an appropriate tool to investigate dorsolateral prefrontal circuit connectivity in MS because previous studies have highlighted the directional information flow of information in this region (Au Duong et al., 2005; Jahfari et al., 2011).

Dobryakova and colleagues have shown that effective connectivity (EC) between the left DLPFC and posterior cingulate cortex correlated positively with task performance in a group of 14 people with primary progressive MS undergoing an attentionally‐demanding (Stroop) task during fMRI (Dobryakova, Rocca, Valsasina, DeLuca, & Filippi, 2017). However, so far there has been no direct evidence to show disrupted EC measured by GCA on resting‐state fMRI data within the dorsolateral prefrontal circuit underlies cognitive performance in MS.

In this study, we hypothesised that EC within the dorsolateral prefrontal circuit, but not the analogous frontal motor circuit, would correlate with cognitive performance, as measured using the paced auditory serial addition test (PASAT) (Gronwall, 1977). PASAT is a non‐specific test of cognition that has been widely applied in MS, with performance being predominantly dependent on processing speed, working memory and sustained attention. In addition, we hypothesised that EC would explain the observed relationships between structural alterations in components of the circuit, including macrostructural (atrophy and white matter hyperintensities volume) and microstructural (radial diffusivity [RD]) integrity of white matter tracts connecting brain regions within the dorsolateral prefrontal circuit, and PASAT performance. RD was chosen to quantify the microstructural damage because of the predominant role of RD in reflecting the subtle pathological changes in MS (Liu, Shu, Duan, & Li, 2011). In addition, we aimed to test stability of the EC measurements in a group of people with MS who underwent repeat MRI scans, and to demonstrate the replicability of key results by repeating the main analyses in an independent cohort of people with MS.

## 2 MATERIALS AND METHODS

### 2.1 Participants

The study population included pooled data from two separate prospective single‐centre observational studies (Study A and Study B) undertaken between 2012 and 2016, with the two cohorts having no overlap. In both studies, people with clinically‐definite diagnoses of RRMS, SPMS or CIS according to the McDonald criteria (2010 revision) (Polman et al., 2011) were recruited from the Neurology MS Clinic based at Nottingham University Hospitals NHS Trust while healthy controls (HCs) were recruited via posters placed on approved noticeboards. The inclusion criteria for both studies are summarised in Table 1. Participants who were pregnant, had neurological, neurosurgical or psychiatric conditions other than MS or had contraindication for MRI were excluded from both studies' recruitment. Additionally, participants who had relapse or change in medication within 30 days prior to the first visit were excluded from Study A while participants who took any MS disease modifying drug in the past 3 months or systemic steroids in the past 60 days were excluded from Study B. Both studies were reviewed and approved by the UK National Research Ethics Service (Study A: 14/EM/0064; Study B: 10/H0408/37). All participants gave written informed consent. Study reporting is compliant with the STROBE initiative statement (Vandenbroucke et al., 2007).

**TABLE 1:** Inclusion and exclusion criteria for Study A, Study B and Independent replication cohort

	Study A	Study B	Independent replication cohort
Inclusion criteria	**Patients:** Age 18–65 years Diagnosis of clinically‐definite MS or clinically isolated syndrome (CIS) Could speak and write in English Be able to given informed consent	**Patients:** Age 18–65 years Diagnosis of clinically‐definite MS or clinically isolated syndrome (CIS) Could speak and write in English Be able to given informed consent	**Patients:** Diagnosis of MS as recorded in their medical records. Able to speak and understand English. Able to give informed consent.
	**Healthy controls:** Age 18–65 years Were healthy Able to provide informed consent	**Healthy controls:** Age 18–65 years Were healthy Able to provide informed consent	
Exclusion criteria	**Patients:** Had neurological, neurosurgical or psychiatric conditions other than MS Be pregnant Had contraindication to MRI Had relapsed or had changed medication within 30 days prior to the first visit	**Patients:** Age < 18; Pregnant; Other neurological or neurovascular condition or previous neurosurgery Involved in MS therapeutic drug trial Taking MS disease modifying drug or has taken any MS disease Modifying drug in the past 3 months Received systemic steroids in the past 60 days Contraindication to MRI Previous reaction to MRI contrast agent	**Patients:** Recent diagnosis of MS, within the last 3 months. Currently in an MS relapse, or have had a relapse in the past 6 weeks Currently taking part in other studies which involve the assessment of mood disorders. Any contraindication to MRI Aged under 16 years Suffer from claustrophobia Pregnant

### 2.2 Cognitive and clinical assessment

We used the Paced Auditory Serial Addition Test with 3‐s stimulus (PASAT‐3) (Gronwall, 1977) as a measure of cognitive performance including information processing speed, working memory and sustained attention. PASAT has been widely used as a measure of cognitive function in people with MS and at the time of data acquisition was used in a number of test batteries in this setting (Benedict et al., 2002; Cutter et al., 1999). The standard pre‐recorded version of the PASAT‐3 (Form A), including the 10‐item practice trial, was administered by a trained, experienced investigator in both Study A and B. PASAT‐3 scores were expressed as Z‐scores using normative data from 385 healthy volunteers on the basis of age, gender, and level of education (Ozakbas et al., 2016), with lower Z‐scores indicating poorer PASAT‐3 performance. The MS group was divided into two subgroups: (a) ‘normal or good PASAT‐3 performance’ (PASAT‐3 Z‐score > −1.5) and (b) ‘poor PASAT‐3 performance’ (PASAT‐3 Z‐score < −1.5) (Matias‐Guiu et al., 2018). To allow characterisation of these groups for burden of motor disability, fine motor and locomotor performance were quantified using the 9‐hole peg test and 25‐ft timed walk respectively (both components of the MS Functional Composite (Cutter et al., 1999)). Handedness was assessed by asking participants what hand they used to write with, or what hand is used more frequently in activities of daily living.

### 2.3 MRI acquisition and quality assessment protocol

All participants underwent MRI scan on a single MRI platform (3T Discovery MR750, General Electric Healthcare; Milwaukee, WI) with a protocol including structural T1‐weighted images, fluid attenuated inversion recovery (FLAIR), resting state fMRI and diffusion tensor imaging (DTI). No MRI software or hardware upgrade occurred between study A and B. The scanning protocols used for Studies A and B were as follows: (a) T1‐weighted axial fast‐spoiled gradient echo (identical sequence used for both studies: FSPGR, TR/TE/TI: 8200/3.1/900 ms, FOV: 256 mm, matrix: 256 × 256 × 156, voxel size: 1 × 1 × 1 mm); (b) Axial T2 FLAIR (identical sequence used for both studies: TR/TE/TI: 8000/120/2250 ms, FOV: 235 mm, matrix: 512 × 512 × 46, voxel size: 0.46 × 0.46 × 3 mm); (c) DTI (Study A: single‐shot diffusion weighted EPI with 4 b0 volumes and 32 diffusion weighted volumes, matrix: 128 × 128 × 66, voxel size: 2 × 2 × 2 mm; Study B: Single‐shot diffusion weighted EPI with 1 b0 volume and 30 diffusion weighted volumes, matrix: 102 × 102 × 46, voxel size: 2.2 × 2.2 × 2.2 mm); (4) fMRI (Study A: TR/TE:2200/36 ms, FA: 80°, matrix: 64 × 64 × 37, voxel size: 3.75 × 3.75 × 3.6 mm, 180 volumes; Study B: Resting‐state fMRI: TR/TE: 2000/40 ms, FA: 90°, matrix: 64 × 64 × 35, voxel size: 3.75 × 3.75 × 3.6 mm, 170 volumes). The first 10 volumes of fMRI data were automatically deleted during data acquisition resulting in 170 volumes for Study A and 160 volumes for Study B.

Automated quality control software MRIQC (https://mriqc.readthedocs.io/en/stable/) was used to identify possible artefacts on resting‐state fMRI and T1‐weighted images resulting from micro‐motion. Outliers on any image‐quality metric scatterplot generated by MRIQC were identified and excluded from all further analysis. Pre‐processing of resting‐state fMRI data included primary head motion correction via realignment to the middle volume (FSL‐MCFLIRT), slice timing correction, brain extraction (FSL‐BET) and spatial smoothing using 5 mm FWHM. Subsequently, we used ICA‐based Automatic Removal of Motion Artefacts (ICA‐AROMA) for automatically detecting and removing motion‐related artefacts. We then applied a high‐pass temporal filter and removed signal from white matter and cerebrospinal fluid.

### 2.4 GCA

**FIGURE 1:**
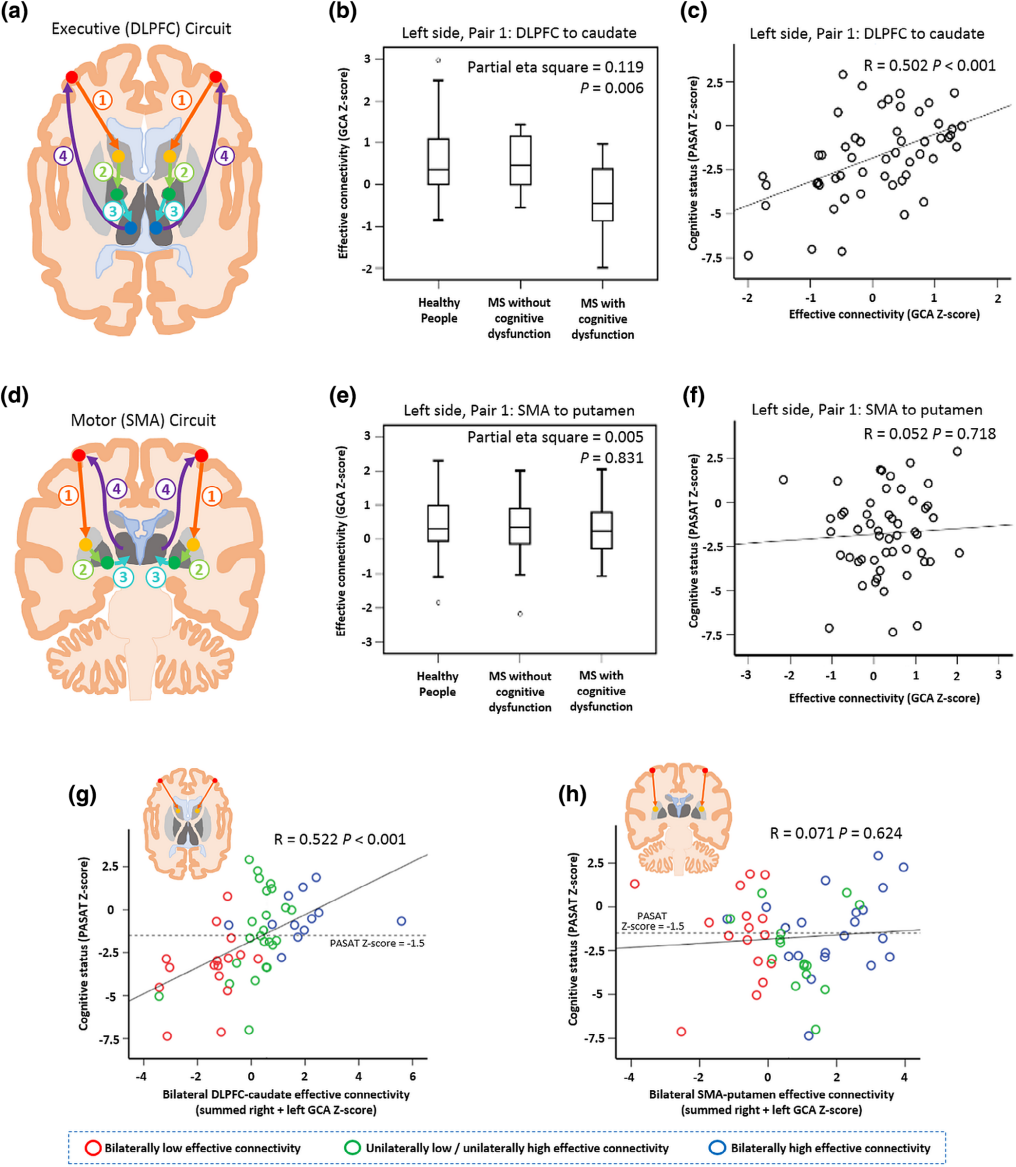
EC within the dorsolateral prefrontal circuit and frontal motor circuit. (a) Schematic illustration showing seed locations for the DLPFC (red dot), caudate (yellow dot), globus pallidus (green dot) and thalamus (blue dot) with numbered arrows indicating the pairwise EC analyses performed. (b) Boxplot demonstrating the significant group difference in left DLPFC‐caudate (pair 1) EC between healthy people, people with MS with normal PASAT‐3 performance and people with MS with impaired PASAT‐3 performance. (c) Scatterplot showing positive correlation between PASAT‐3 Z‐score and DLPFC‐caudate (pair 1) EC in people with MS (correlation controlled for age and gender). (d) Frontal motor circuit: Schematic illustration showing seed locations for the SMA (red dot), putamen (yellow dot), globus pallidus (green dot) and thalamus (blue dot) with numbered arrows indicating the pairwise EC analyses performed. (e) and (f) Boxplot and scatterplot for SMA‐putamen EC corresponding to those shown in (b) and (c). (g) and (h) Correlation between cognition and averaged bilateral EC within (g) the dorsolateral prefrontal circuit and (h) the frontal motor circuit for people with MS. Participants were grouped into those with bilaterally low EC (red circles), unilateral low/unilateral high EC (green circles) and bilateral high EC (blue circles)

A web‐interface platform for large‐scale, automated synthesis of fMRI data (http://neurosynth.org/) was used to determine MNI coordinates of seed regions for GCA. A term‐based meta‐analysis of studies in the NeuroSynth database was automatically run for the seed regions. This approach allowed us to base our placement of regions‐of‐interest (ROI) on a statistical consensus across the literature. MNI coordinates of the voxel with the largest Z‐score, which was the most frequently reported in all studies in the term‐based meta‐analysis, were used to define the centre of the ROI. Six millimetre radius spheres (Iwabuchi et al., 2017) were centred on DLPFC (MNI coordinates: left [−30,43,22]; right [38,37,22]), caudate head (left [−11,13,10]; right [14,13,11]), pallidum (left [−26,0,6]; right [26,6,4]) and thalamus (left [−9,−17,6]; right [10,−19,6]) bilaterally as seed regions for the GCA of the dorsolateral prefrontal circuit (Figure 1a). Bivariate first‐order coefficient‐based voxelwise GCA was performed using REST software (http://www.restfmri.net), which we used for its ability to output the residual‐based F and transformed Z statistics. The GCA was conducted for each hemisphere separately with each hemisphere having four pairs of ROIs (Figure 1a). To test for specificity of the relationship between PASAT‐3 performance status and EC of the dorsolateral prefrontal circuit, we repeated the analysis using seeds positioned in the frontal motor circuit (supplementary motor area [SMA], putamen, globus pallidus and thalamus) using the same approach (Figure 1d).

### 2.5 Stability of EC measurement in people with MS

To allow investigation of stability of EC metrics, a subset of MS participants (*n* = 17) in the Study B were invited for repeat MRI scan with the same scanner and fMRI protocol with 6‐month target interval after the first scan, as described previously (Welton, Constantinescu, Auer, & Dineen, 2020). fMRI quality assessment and processing performed as described above. Intraclass correlation of GCA Z values was tested (two‐way random effects, single measures, consistency) and classified as ‘poor’ (<0.5), ‘moderate’ (0.5–0.75), ‘good’ (0.75–0.9) or ‘excellent’ (0.9–1.00) according to guidelines for reporting of ICCs (Koo & Li, 2016).

### 2.6 Quantification of macrostructural damage within the dorsolateral prefrontal circuit

Quantification of macrostructural damage included volumetric measurements of cortical and subcortical grey matter structures and T2‐hyperintense lesion volumes within the dorsolateral prefrontal circuit. To quantify volumes of subcortical grey matter structures (caudate, pallidum and thalamus) from each hemisphere we used FIRST in FSL (version 5.0.11) (Patenaude, Smith, Kennedy, & Jenkinson, 2011). To quantify cortical thinning we calculated the local gyrification index (LGI) which estimates cortical folding by expressing the amount of cortex buried within sulcal folds relative to the visible cortex across the 3D cortical surface (Schaer et al., 2008). Reduced LGI reflects cortical thinning on the basis that degenerative cortex shows reduced folding, and has shown better test–retest reliability than cortical thickness measurement (Madan & Kensinger, 2017). Cortical surface identification, parcellation and LGI were obtained using FreeSurfer v6.0.0 (Fischl & Dale, 2000; Schaer et al., 2008). LGI of the middle frontal gyrus, superior frontal gyrus, superior frontal sulcus, middle frontal sulcus and inferior frontal sulcus labels in the Destrieux Atlas were averaged to give DLPFC LGI for each hemisphere (Yamagishi et al., 2016).

T2‐hyperintense lesions (T2HL) were segmented on FLAIR images for the whole brain by a trained investigator (D.M.) using NeuRoi (https://www.nottingham.ac.uk/research/groups/clinicalneurology/neuroi.aspx). T2‐hyperintense lesion volumes were calculated using FSL (version 5.0.11) and were normalised to whole brain volume.

### 2.7 Quantification of microstructural damage within the dorsolateral prefrontal circuit

Quantification of microstructural damage included measurements of radial diffusivity (RD) of white matter tracts (WMTs) connecting brain regions within the dorsolateral prefrontal circuit. We chose RD as the DTI metric to reflect microstructural damage because it offers a metric that is reported it index demyelination (Song et al., 2005). Pre‐processing of DTI data sets from the participants with MS was performed using the diffusion toolbox of FSL (version 5.0.11) (Behrens et al., 2003).

To generate a high quality mask for WMTs connecting components of the dorsolateral prefrontal circuit, we used pre‐processed DTI data of 88 healthy subjects (age range: 31–35, gender‐matched to our MS population) from the Human Connectome Project (HCP) Young Adult Cohort (http://www.humanconnectome.org) with acquisition parameters as published previously (WU‐Minn, 2017). The probabilistic tractography algorithm provided by FSL (version 5.0.11) (Behrens et al., 2003; Behrens, Berg, Jbabdi, Rushworth, & Woolrich, 2007) was used to reconstruct WMTs connecting pairs of ROI which showed lower EC in our MS cohort compared to the control group. We co‐registered each HCP participant's segmented masks of brain regions within dorsolateral prefrontal circuit (bilateral DLPFC, caudate, pallidum and thalamus) to the DTI data and used these co‐registered seed masks for tractography analysis. Each HCP participant's reconstructed WMTs were normalised to the total number of samples making it from seed to target and thresholded at the level of 0.005%. All HCP participants' normalised and thresholded WMTs were averaged and registered to MNI152 space to create an atlas of WMTs within the dorsolateral prefrontal circuit. Standard tract‐based spatial statistics (TBSS) procedure using the generated skeleton template was applied to the DTI data from our MS and control groups to provide an aligned white matter skeleton for each participant (Alhilali, Yaeger, Collins, & Fakhran, 2014; Smith et al., 2006). The masks of reconstructed WMTs within the dorsolateral prefrontal circuit derived from the HCP data were overlaid onto each participant's skeletonised RD, and mean RD value for the skeletonised WMT was extracted.

### 2.8 Replication of results in an independent MS population

We sought to replicate key findings of the study by repeating the methodological steps above using data from an independent group of people with MS (Study C: 10/H408/10, *n* = 14) recruited from a study which investigated depression in MS. This cohort had no overlap with Studies A or B. Due to the primary question being addressed in study C which related to mood disorder in MS, the inclusion and exclusion criteria varied slightly from study A and B (Table 1) and the MRI scans were performed on a different 3T MRI scanner (Supplementary materials).

### 2.9 Statistical analysis

One‐way analysis of variance and χ^2^ tests in SPSS (version 24; SPSS, Chicago) were used to compare demographics between healthy people, people with MS with poor PASAT‐3 performance and people with MS with normal or good PASAT‐3 performance. Statistical significance was defined as *p* < .05.

To investigate EC within the dorsolateral prefrontal circuit and its association with PASAT‐3 performance, we extracted mean GCA coefficients from brain regions of dorsolateral prefrontal circuit from Z‐transformed X‐to‐Y GCA maps to identify the directional influence between eight pairs of ROIs. We overlapped the mask of downstream ROI onto the GCA maps of the upstream seed region to compute GCA Z‐score for each pair of ROIs. For example, the EC from left DLPFC to left caudate was calculated by extracting the GCA Z‐score of left caudate from the GCA map of left DLPFC. Therefore, EC between each pair of ROIs was indexed as the Z‐score of the GCA value. All statistical tests were conducted in SPSS and controlled for age and gender. Benjamini‐Hochberg procedure was applied to correct for the false discovery rate (FDR) (Benjamini & Hochberg, 1995). The general linear model univariate analysis was used to compare the difference of GCA Z‐score among healthy people, people with MS with impaired PASAT‐3 performance and people with MS without impaired PASAT‐3 performance. Statistical significance level was at FDR‐corrected *p* < .05. Partial correlation analyses were conducted to investigate the association between PASAT‐3 Z‐score and GCA Z‐score in people with MS, with significance defined as FDR‐corrected *p* < .05. To test for specificity of the relationship between PASAT‐3 performance status and EC of the dorsolateral prefrontal circuit, these statistical analyses were repeated using the EC measures extracted from the frontal motor circuit treating age and gender as covariates of no interest.

To investigate whether there was an additive effect of bilaterally reduced EC from DLPFC‐to‐caudate on PASAT‐3 score, we ran post‐hoc correlation analysis of the summed (right + left) DLPFC‐caudate GCA Z‐score with PASAT‐3 Z‐score, and conducted three‐way group analysis of PASAT‐3 performance using χ^2^ test by classifying people with MS as having (a) bilaterally low (b) unilaterally low or unilaterally high, or (c) bilaterally high DLPFC‐caudate EC. Classification into low or high EC was based on median split of DLPFC‐caudate GCA Z‐score across the cohort, calculated separately for each side.

Principal component analysis (PCA) in SPSS (version 24) was used to create a factor score to reflect overall structural damage between two regions within the dorsolateral prefrontal circuit. Given that we had several structural imaging metrics, including the grey matter quantification of the brain regions (e.g., normalised volume of caudate, pallidum, thalamus; LGI of DLPFC) and RD of WMTs connecting two brain regions within the dorsolateral prefrontal circuit, to reflect the structural damage of the dorsolateral prefrontal circuit we used factor analysis with PCA method in SPSS (version 24) for data reduction purposes. Factors with eigenvalue exceeding 1.0 were extracted and the factor score for each participant (a linear combination of structural damage weighted by factor loadings) was computed using regression method in SPSS. We used factor scores to reflect overall structural damage between two regions within the dorsolateral prefrontal circuit.

We used Spearman correlation analysis in SPSS (version 24) to investigate the associations between EC (GCA Z‐score), structural integrity (factor score) of dorsolateral prefrontal circuit and PASAT‐3 Z‐score. Statistical significance was defined as FDR‐corrected *p* < .05. Left and right sides were analysed separately.

We performed mediation analysis to investigate whether EC changes of dorsolateral prefrontal circuit mediate the effect of structural changes of dorsolateral prefrontal circuit and PASAT‐3 performance using the PROCESS v3.1 macro (http://www.processmacro.org/index.html) for SPSS 24. The significance of indirect effects was tested using bootstrapping with 5,000 replications. Mediation is accepted as having occurred if the indirect effect (x*y) is statistically significant.

## 3 RESULTS

Sixty‐five people with MS and 47 healthy people were included in the initial pooled study cohort. After image data quality control for motion and artefacts, 52 people with MS (mean age ± *SD*: 46.2 ± 11.44; 38 female [73.1%]) and 36 healthy people (mean age ± *SD*: 41.9 ± 12.59; 26 female [72.2%]) were included for further analysis (Table 2). No significant difference of demographical or clinical characteristics was found between PASAT‐performance‐impaired MS and PASAT‐performance‐unimpaired MS. After data quality control, 12 people with MS (Table 2) were included in the replication cohort. There was no significant difference of demographical or clinical characteristics between MS participants in the replication cohort and those in the pooled study cohort.

**TABLE 2:** Demographics and clinical information of participants in the pooled study cohort and independent replication cohort

	Healthy people, *n* = 36	People with multiple sclerosis (MS), *n* = 52		*p* ^a^	Independent replication cohort (*n* = 12)
		Normal PASAT‐3 performance, *n* = 23	Impaired PASAT‐3 performance, *n* = 29		
Age (years), mean ± *SD*	41.9 ± 12.6	45.3 ± 11.3	46.9 ± 11.7	.238^b^	46.1 ± 10.9
Female sex, *n* (%)	26 (72.2%)	16 (69.6%)	22 (75.9%)	.876^c^	10 (83.3)
Right handedness, *n* (%)	34 (94.4%)	18 (78.3%)	26 (89.7%)	.158^c^	–
Education (years), mean ± *SD*	–	14.2 ± 2.9	13.2 ± 2.3	.217^d^	13.7 ± 2.3
Disease duration (years), mean ± *SD*	–	–	14.8 ± 10.5	.964^d^	–
MS subtype:				.664^c^	–
Secondary progressive MS, *n* (%)	–	6 (26.1%)	11 (37.9%)		
Relapsing remitting MS, *n* (%)	–	16 (69.6%)	17 (58.6%)		
Clinically isolated syndrome, *n* (%)	–	1 (4.3%)	1 (3.4%)		
Z score of timed 25‐ft walk	–	−0.05 ± 0.57	0.34 ± 2.98	.522	–
Z score of 9‐hole peg test	–	−0.22 ± 0.90	−0.57 ± 1.41	.293	–
Beck depression inventory‐II score	–	13.7 ± 11.7	12.9 ± 9.9	.786	–

^a^ All statistical analyses were conducted in the main study cohort (Study A and Study B).

^b^ ANOVA test was used for statistical analysis.

^c^ χ^2^ test was used for statistical analysis.

^d^ Independent t‐test was used for statistical analysis.

### 3.1 EC in the dorsolateral prefrontal circuit is reduced in people with MS with impaired PASAT‐3 performance

Significant differences in DLPFC‐to‐caudate EC were found between healthy people (mean ± *SD*: left: 0.54 ± 0.85; right: 0.53 ± 0.74), PASAT‐performance‐unimpaired people with MS (mean ± *SD*: left: 0.54 ± 0.66; right: 0.48 ± 1.05) and PASAT‐performance‐impaired people with MS (mean ± *SD*: left: −0.34 ± 0.83; right: −0.31 ± 1.10) in both left (Figure 1b, Partial Eta Squared = 0.119, *p* = .006) and right hemispheres (Partial Eta Squared = 0.084, *p* = .029) while treating age and gender as covariates of no interest (Figure 1a, orange arrow; Table 3). When including T2HL volume as an additional covariate of no interest, these differences remained significant in both left (Partial Eta Squared = 0.116, *p* = .007) and right hemispheres (Partial Eta Squared = 0.057, *p* = .047). No significant differences in other pairs of ROIs within the dorsolateral prefrontal circuit (caudate‐pallidum, pallidum‐thalamus, thalamus‐DLPFC) were identified among these groups. Post hoc *t* tests showed people with MS with PASAT‐3 performance impairment (*n* = 29) had decreased DLPFC‐to‐caudate EC in both left (Partial Eta Squared = 0.157, *p* = .005) and right (Partial Eta Squared = 0.098, *p* = .029) hemispheres compared to those without PASAT‐3 performance impairment (*n* = 23).

**TABLE 3:** Imaging metrics of participants in the pooled study cohort and independent replication cohort

	Healthy people, *n* = 36	People with multiple sclerosis (MS), *n* = 52		*p* ^a^	Independent replication cohort (*n* = 12)
		Normal PASAT‐3 performance, *n* = 23	Impaired PASAT‐3 performance, *n* = 29		
Normalised T2 hyperintense lesion volume (%), mean ± *SD*	–	0.01 ± 0.01	0.03 ± 0.03	.005*^,^ ^b^	0.02 ± 0.02
Brain parenchymal fraction, mean ± *SD*	–	0.79 ± 0.05	0.78 ± 0.05	.239^b^	0.78 ± 0.05
Left dorsolateral prefrontal cortex local gyrification index, mean ± *SD*	2.75 ± 0.13	2.74 ± 0.12	2.72 ± 0.13	.725^c^	2.78 ± 0.13
Right dorsolateral prefrontal cortex local gyrification index, mean ± *SD*	2.79 ± 0.15	2.75 ± 0.10	2.75 ± 0.14	.474^c^	2.73 ± 0.12
Normalised volume of left caudate (%), mean ± *SD*	0.34 ± 0.03	0.31 ± 0.03	0.29 ± 0.04	<.001*^,^ ^c^	0.34 ± 0.09
Normalised volume of right caudate (%), mean ± *SD*	0.36 ± 0.04	0.33 ± 0.03	0.33 ± 0.07	.063^c^	0.36 ± 0.11
Radial diffusivity of white matter tract connecting left dorsolateral prefrontal cortex and caudate (× 10^−3^ mm^2^/s), mean ± *SD*	0.65 ± 0.07	0.77 ± 0.14	0.86 ± 0.15	<.001*^,^ ^c^	0.82 ± 0.11
Radial diffusivity of white matter tract connecting right dorsolateral prefrontal cortex and caudate (× 10^−3^ mm^2^/s), mean ± *SD*	0.64 ± 0.08	0.86 ± 0.25	0.93 ± 0.23	<.001*^,^ ^c^	0.85 ± 0.21
Left DLPFC‐to‐caudate effective connectivity (EC)	0.54 ± 0.85	0.54 ± 0.66	−0.34 ± 0.83	.006*	−0.59 ± 1.54
Right DLPFC‐to‐caudate EC	0.53 ± 0.74	0.48 ± 1.05	−0.31 ± 1.10	.029*	0.13 ± 1.54
Left supplementary motor area (SMA)‐to‐putamen EC	0.43 ± 0.87	0.27 ± 0.95	0.29 ± 0.74	.831	–
Right supplementary motor area (SMA)‐to‐putamen EC	0.41 ± 0.80	0.40 ± 1.22	0.52 ± 0.84	.725	–

^a^ All statistical analyses were conducted in the main study cohort (Study A and study B).

^b^ Independent *t*‐test was used for statistical analysis.

^c^ ANOVA test was used for statistical analysis.

*Significant level at *p* < .05.

### 3.2 EC in the dorsolateral prefrontal circuit, but not the motor circuit, correlates with PASAT‐3 performance

In people with MS, PASAT‐3 Z‐score was significantly correlated with DLPFC‐to‐caudate EC in both left (*r* = .502, *p* < .001; Figure 1c) and right (*r* = .371, *p* = .008) hemispheres while controlling for age and gender. After additionally controlling for T2HL volume, correlation between PASAT‐3 Z score and DLFPC‐to‐caudate EC remained significant in both left (*r* = 0.495, *p* < .001) and right (*r* = .328, *p* = .021) hemispheres. No significant correlation was identified between PASAT‐3 Z‐score and caudate‐pallidum, pallidum‐thalamus or thalamus‐DLPFC EC for left or right hemisphere, controlling for age and gender.

Bilaterally reduced EC from DLPFC to caudate had an additive effect on PASAT‐3 score. Bilaterally summed DLPFC‐caudate GCA Z‐score significantly correlated with PASAT‐3 Z‐score (*r* = .522, *p* < .001, controlled for age and gender; Figure 1g). In people with MS (*n* = 52), 16 (31%) had bilaterally low DLPFC‐to‐caudate EC, 24 (46%) had unilaterally low/unilaterally high DLPFC‐caudate EC, and 12 (23%) bilaterally high DLPFC‐caudate EC. There was a significant group difference in PASAT‐3 score between these groups (Partial Eta Squared = 0.241, *p* = .002), with the bilaterally low DLPFC‐caudate EC group having the poorest PASAT‐3 performance.

To test for specificity of the relationship between reduced EC in the dorsolateral prefrontal circuit and PASAT‐3 performance group, we repeated our analysis in the frontal motor circuit. The SMA‐to‐putamen EC was not significantly different among HCs, people with MS without impaired PASAT‐3 performance and people with MS with PASAT‐3 performance dysfunction for either the left (Partial Eta Squared = 0.005; *p* = .831, Figure 1e) or right hemispheres (Partial Eta Squared = 0.008; *p* = .725), and was not significantly correlated with PASAT‐3 Z‐score in people with MS (left hemisphere: *r* = .052; *p* = .718, Figure 1f; right hemisphere: *r* = .071; *p* = .626; Table 3). We repeated the analysis to test for correlation between PASAT‐3 Z‐score and summed SMA‐putamen GCA Z‐score across both hemispheres controlling for age and gender but found none (*r* = .071, *p* = .624, Figure 1h).

### 3.3 Stability of effective connectivity measures

**FIGURE 2:**
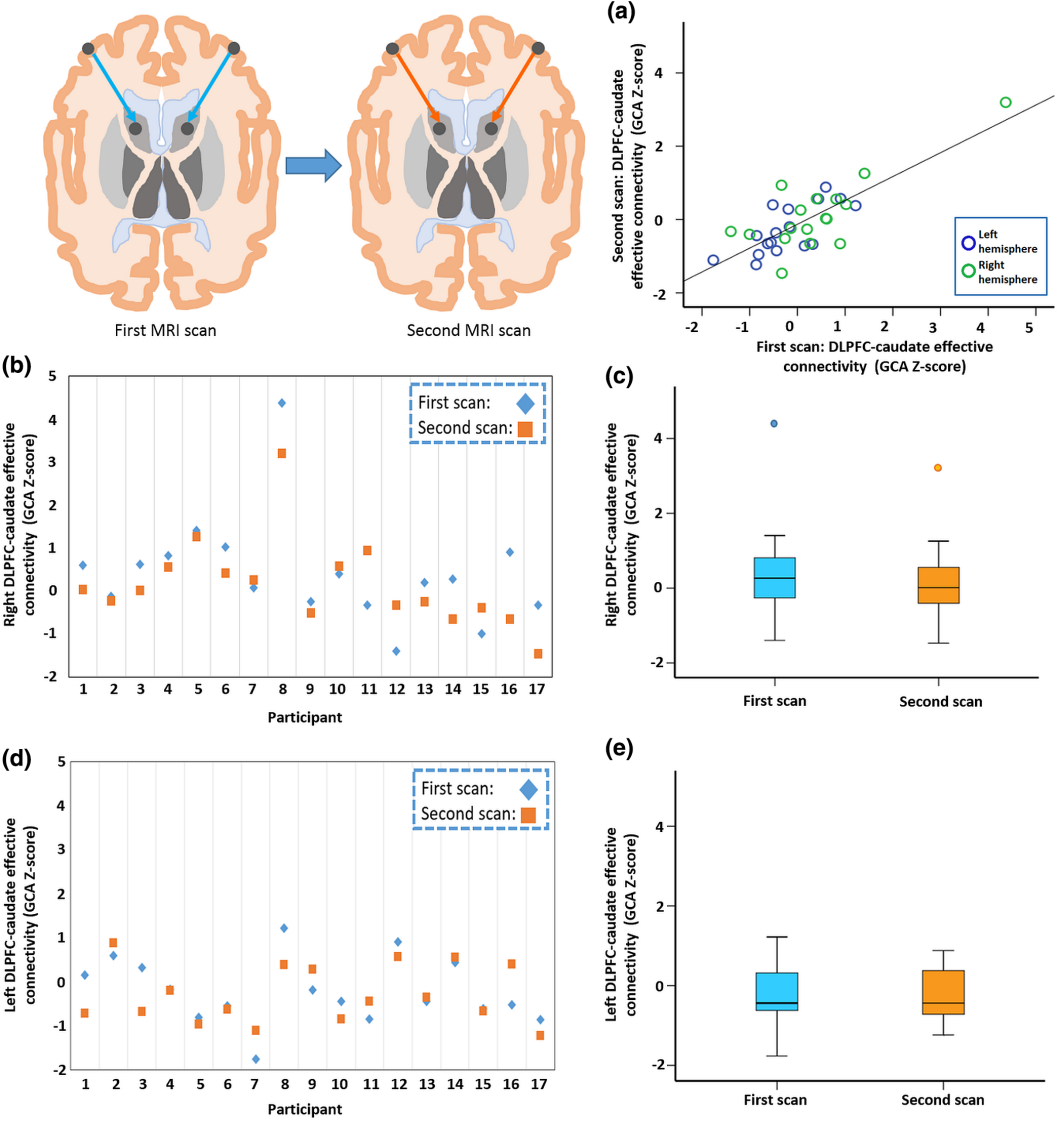
Stability of EC from DLPFC to caudate in people with MS. (a) Scatterplot of the DLPFC‐caudate EC measures for left (blue circles) and right (green circles) hemispheres. (b) Plot of right DLPFC‐caudate EC from the first (blue diamond) and second (orange square) scans for the 17 individual participants with MS who accepted the invitation to attend for the repeat assessment, and (c) the boxplots summarising the whole group results demonstrating absence of group difference between the first and second scan. The respective plots for the left side are shown in (d) and (e), displayed on the same axes as (b) and (c)

The mean interval between first and second scans for the 17 people with MS who attended for a repeat fMRI scan with good data quality was 208 days (*SD*: 53). Paired *t* test showed no significant difference between baseline and follow‐up for left (*p* = .606) or right (*p* = .172) DLPFC‐caudate EC. Intraclass correlation coefficient for EC between DLPFC and caudate was ‘good’ for the right (R_ICC_ = 0.775) and ‘moderate’ for the left (R_ICC_ = 0.711) (Figure 2).

### 3.4 Structural damage in the anterior limb of the dorsolateral prefrontal circuit is associated with EC changes and PASAT‐3 performance in MS

In people with MS, PCA analysis yielded a single significant model for structural damage of the anterior limb of the dorsolateral prefrontal circuit on each side that comprised DLPFC LGI, normalised volume of caudate and RD of WMTs connecting DLPFC to caudate (left side: eigenvalue = 1.61, accounting for 53.9% of the variance; right side: eigenvalue = 1.39, accounting for 44.8% of the variance). Structural damage of the anterior limb of the dorsolateral prefrontal circuit (factor score of the significant PCA model) correlated with DLPFC‐caudate EC in both left (*r* = .358, *p* = .009) and right (*r* = .315, *p* = .023) hemisphere (FDR‐corrected *p* = .05). Structural damage in the left (*r* = .402, *p* = .003) and right (*r* = .361, *p* = .008) hemisphere also correlated with PASAT‐3 Z‐score (FDR‐corrected *p* = .05).

### 3.5 Effective connectivity between DLPFC and caudate mediates the relationship between structural changes and PASAT‐3 performance

**FIGURE 3:**
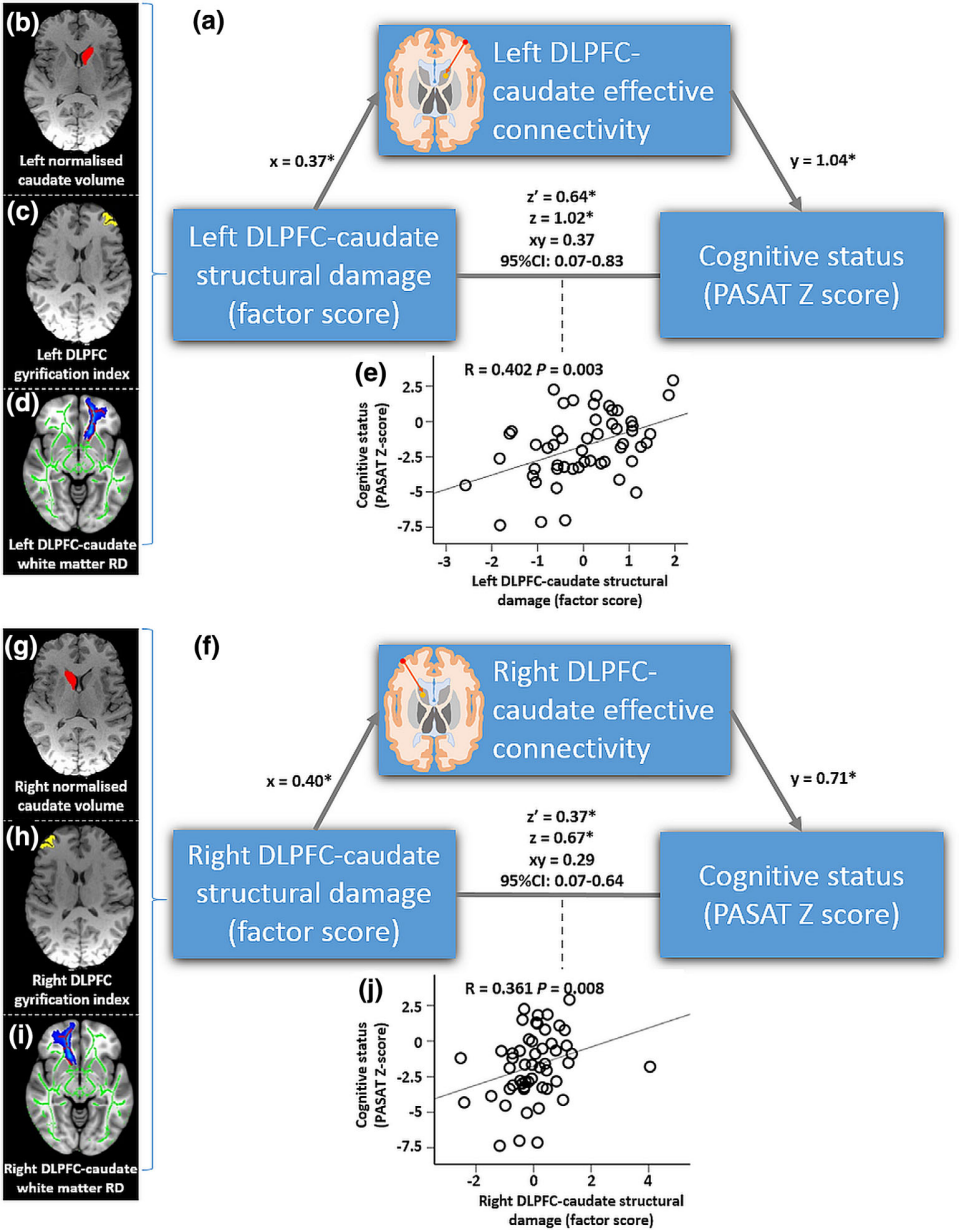
(a) Mediation model testing whether altered EC mediates the association between PASAT‐3‐performance‐relevant structural damage in the dorsolateral prefrontal circuit and PASAT‐3 performance for the left hemisphere. The left DLPFC‐caudate structural damage (factor score) comprised the left DLPFC LGI, the radial diffusivity (RD) of WMTs connecting DLPFC and caudate and the left caudate volume. In the model, x is the unstandardised regression coefficient of the association between DLPFC‐caudate EC and structural damage, y is the unstandardised regression coefficient of the association between DLPFC‐caudate EC and cognition while also controlling for DLPFC‐caudate EC, xy is the indirect effect, z is the total effect and z′ is the direct effect. The mediating effect of the left DLPFC‐caudate EC was significant in this relationship. (c, d) Corresponding mediation model and components for the right hemisphere

For left and right sides (analysed separately) DLPFC‐caudate GCA Z‐score was selected as the potential mediator of the association between structural damage in the dorsolateral prefrontal circuit and PASAT‐3 performance given that it significantly correlated with both. Left DLPFC‐caudate GCA Z‐score mediated the association between PASAT‐3 Z‐score and structural damage composite score (xy = 0.37 [95%CI: 0.07–0.83], Figure 3b). Right DLPFC‐caudate Z‐score mediated the association between PASAT‐3 Z‐score and structural damage composite score (xy = 0.29 [95%CI 0.07–0.64], Figure 3d).

### 3.6 Key findings are replicable in an independent MS cohort

After data quality control, 12 people with MS (Table 2) were included in the replication cohort. In this modest data set, we attempted to replicate key findings in the above analyses. Significant differences in DLPFC‐to‐caudate EC were found between PASAT‐performance‐unimpaired people with MS (*n* = 9, mean ± *SD*: left: 0.06 ± 1.04; right: 0.77 ± 0.85) and PASAT‐performance‐impaired people with MS (*n* = 3, mean ± *SD*: left: −2.58 ± 0.95; right: −1.80 ± 1.60) in both left (Partial Eta Squared = 0.626, *p* = .006) and right hemispheres (Partial Eta Squared = 0.667, *p* = .004) while treating age and gender as covariates of no interest. PASAT‐3 Z‐score was significantly correlated with DLPFC‐to‐caudate EC in both left (*r* = .701, *p* = .024) and right (*r* = .808, *p* = .005) hemispheres while controlling for age and gender.

## 4 DISCUSSION

In this hypothesis‐driven study we used multimodal imaging to establish a role for altered structural and functional integrity of dorsolateral prefrontal circuit in people with MS with impaired PASAT‐3 performance. Our first hypothesis, that EC within the dorsolateral prefrontal circuit would correlate with PASAT‐3 performance is supported by our findings. Key results in this regard are the finding that (a) people with MS with PASAT‐3 performance impairment showed decreased DLPFC‐to‐caudate EC compared to people with MS without PASAT‐3 performance impairment and healthy people, (b) impaired top‐down DLPFC‐to‐caudate EC in both left and right hemispheres correlated with PASAT‐3 performance in people with MS, and (c) people with MS with reduced DLPFC‐to‐caudate EC bilaterally had worse PASAT‐3 performance than those with only unilateral reduction in EC. Notably, the relationships between the DLPFC‐to‐caudate EC and PASAT‐3 performance scores were slightly stronger on the left than the right, which may relate to asymmetries in task processing shown by previous fMRI studies demonstrating greater left sided activations during the PASAT task (Audoin et al., 2005). However, given that we did not have an objective assessment of handedness and we did not exclude left‐handedness participants, we cannot fully exclude the possibility that this specific finding is confound by handedness and thus this result should be interpreted cautiously. Measurements of DLPFC‐to‐caudate EC were stable over an average 8‐months interval in people with MS which may be relevant for use of this metric in future cognitive intervention studies. Associations between PASAT performance and EC in the frontal direct motor circuit were not found, supporting a specific role for the dorsolateral prefrontal circuit for cognitive processes related to PASAT performance (Middleton & Strick, 2000).

The second hypothesis tested, that EC would explain observed relationships between structural alterations in components of the circuit and PASAT‐3 performance, is also supported by our findings. The key supportive results here are (a) lower DLPFC‐to‐caudate EC was related to distinct ipsilateral structural damage (DLPFC gyrification index, caudate volume and microstructural damage of white matter tract connecting right DLPFC and caudate); and (b) DLPFC‐to‐caudate EC partially mediated the link between combined structural damage and PASAT‐3 performance.

Our results are consistent with, and build on, previous studies identifying functional connectivity changes as being relevant to cognition in people with MS. In a study of 26 individuals with MS and a similar number of controls, Leavitt and colleagues used an fMRI task of information processing speed to show that people with MS had more connections from multiple regions to the frontal cortices than controls, which they interpreted as indicating greater neural recruitment to maintain performance in the MS group (Leavitt, Wylie, Genova, Chiaravalloti, & DeLuca, 2012). Dobryakova et al. showed correlation of EC between the left DLPFC and posterior cingulate cortex with task performance during an attentionally‐demanding task fMRI in a small group of people with primary progressive MS (Dobryakova et al., 2017). This work was extended to include other MS phenotypes (relapsing–remitting, benign and secondary progressive MS), demonstrating that all MS phenotypes had impaired EC from the right DLPFC (Dobryakova et al., 2016). Our study varies significantly from these in that we establish EC differences without cognitive loading during resting‐state fMRI, and correlate connectivity measures with cognitive performance measured outside of the MRI scanner using the PASAT‐3 (a non‐specific test which includes information processing speed).

Our post hoc analysis revealed that people with MS who had reduced EC in both hemispheres had poorer PASAT‐3 performance than those who had reduced EC in one hemisphere, suggesting that bilateral reduction in DLPFC to caudate EC has a greater impact than unilateral reduction alone. Future studies are needed to identify the cut‐off score of the GCA Z‐value of the DLPFC to caudate and to further investigate whether the status of dorsolateral prefrontal circuit EC can differentiate people with MS with and without impaired PASAT‐3 performance.

A key objective of our study was to characterise the association between structural damage and EC alteration within the dorsolateral prefrontal circuit and PASAT‐3 performance. We found that reduced DLPFC‐to‐caudate EC in left and right hemisphere had distinct association with underlying structural damage, including cortical folding of DLPFC, caudate volume and microstructural damage of WMTs connecting DLPFC and caudate. We found that an index score derived from these three structural parameters showed significant correlation with PASAT‐3 performance. Previous studies have shown regional damage of the dorsolateral prefrontal circuit is detectable in people with MS with information processing speed deficits, including the atrophy of DLPFC, caudate or thalamus (Batista et al., 2012; Nocentini et al., 2014), and our results confirm this association. Additionally, we found a relationship between PASAT‐3 performance and RD in the WMT linking the DLPFC to the caudate which is in line with previous systematic reviews identifying relationships between frontal white matter integrity and cognition in MS (Manca et al., 2018; Welton, Kent, Constantinescu, Auer, & Dineen, 2015). We chose RD as the DTI metric to reflect microstructural damage because it has been reported to be an index of demyelination (Song et al., 2005) and it has been shown that RD may be the most sensitive of the DTI‐derived metrics when estimating microstructural damage in MS (Lipp et al., 2019).

We sought to understand the three‐way associations between structure, EC and PASAT‐3 performance by conducting mediation analysis based on the relationship proposed in the second hypothesis and demonstrate that DLPFC‐to‐caudate EC in both hemispheres partially mediates the association between underlying structural damage and PASAT‐3 performance. While the mediation analysis does not allow inference on causality, our findings suggest that impaired DLPFC‐to‐caudate EC bilaterally may be an intermediary mechanism linking structural damage with PASAT‐3 function. As well as improving our mechanistic understanding of impaired cognitive performance in MS and narrowing the clinico‐radiological paradox (Barkhof, 2002), demonstration of an intermediary role for altered EC could support further studies into therapeutic strategies that improve cognitive performance by inducing functional alterations in the dorsolateral prefrontal circuit through cognitive training or stimulation approaches.

Lack of replication of results is acknowledged to be a major problem in scientific research (Baker, 2016) and in particular for fMRI studies in people with MS (Sumowski et al., 2018). We address this for our main analysis by performing replication of key findings in an independent data set. Despite the replication data set having a small sample size and being scanned on a different MRI system, we demonstrated similar effect sizes in the same direction as those from the main analysis. The generally higher *p*‐values in the validation data set are likely to reflect a degree of under‐powering of the analyses.

A potential criticism of our work is the use of PASAT‐3 as a cognitive measure. PASAT‐3 has been used extensively to detect cognitive impairment in MS and has been incorporated into versions of a number of cognitive and clinical test batteries in MS. However, PASAT‐3 suffers from practice effects and performance may also be impacted by mathematical ability, speech production and anxiety/frustration (Tombaugh, 2006), and consequently has been replaced in many batteries by other tests such as the symbol digit modalities test, which tests information processing speed. The participants in our study underwent PASAT‐3 in a standardised presentation (single administration following the standard practice items) and hence practice effects are unlikely to confound the results, but as we did not record whether participants had undergone PASAT‐3 assessment in the past we cannot fully exclude confounding practice effects. Future work exploring the link between the dorsolateral prefrontal circuit and cognitive performance in MS would benefit from use of neuropsychological tests that have greater specificity for relevant cognitive constructs and are less susceptible to confounding effects.

A further limitation of our work was that we used a pooled data set to test our hypotheses which included minor differences in inclusion/exclusion criteria and MRI acquisition. The replication data set was acquired on a different MRI scanner and using a slightly different protocol to the main analysis but this is not necessarily a weakness; the fact that we identify similar relationships despite differences in the MRI acquisition is supportive that the relationships have a biological basis.

## 5 CONCLUSIONS

Our study shows that reduced EC in the anterior limb of the dorsolateral prefrontal circuit occurs in people with MS who perform poorly in the PASAT‐3, and partially mediates the relationship between structural alterations in this circuit and PASAT‐3 performance. Key results have been replicated in a modest separate cohort of people with MS. We demonstrate specificity of the relationship between PASAT‐3 performance and the dorsolateral prefrontal circuit by showing an absence of any association between PASAT‐3 performance and EC in the analogous and anatomically proximal frontal motor circuit. We show good test–retest reliability of EC measurement in the dorsolateral prefrontal circuit in people with MS over an average 8‐month interval. These findings highlight disconnection of the dorsolateral prefrontal circuit—in particular the DLPFC‐to‐caudate pathway—as a key mechanism for impairment of PASAT‐3 performance and information processing in people with MS. This may therefore be a potential target for further study into therapeutic strategies aimed at maintaining and improving cognitive performance in people with MS through modulation of EC.

## CONFLICT OF INTEREST

All authors declare no conflicts of interest.

## ETHICS STATEMENT

Both studies included in this manuscript were reviewed and approved by the UK National Research Ethics Service (Study A: 14/EM/0064; Study B: 10/H0408/37). All participants gave written informed consent. Study reporting is compliant with the STROBE initiative statement.

## Supporting information


**Supplementary materials S1**. Supplementary materials.

## Data Availability

The data that support the findings of this study are available from the corresponding author upon reasonable request.
