# Supplementary material for: Dorsolateral prefrontal circuit effective connectivity mediates the relationship between white matter structure and PASAT‐3 performance in multiple sclerosis
Source: Hum Brain Mapp. 2020 Oct 19;42(2):495–509. doi: 10.1002/hbm.25239 (PMC7776003; doi:10.1002/hbm.25239)
Supplement: Supplementary file 1 — Supplementary materials S1. Supplementary materials. [file HBM-42-495-s001.pdf]

8. Supplementary Materials

Image acquisition protocol for the independent replication cohort

Scans were performed on a 3T Philips Achieva with 16-channel SENSE head and neck coil. Sequences were as follows:

Whole brain axial FLAIR: TR=1100ms, TE=125ms, TI=2800ms, flip angle: 90º, 512x512x45 matrix, resolution 0.5x0.5x3mm.

Whole brain DTI: Single-shot, spin-echo, echo-planar, diffusion-weighted sequence is used (acquisition matrix 112x112 with in-plane resolution 2x2 mm^2, without any interpolation). A parallel imaging factor of 2 and a partial Fourier factor of 0.7 are employed. Eight b=0 s/mm^2 images are acquired and averaged. Diffusion weighting is applied in 61 evenly spaced directions with b=1000 s/mm^2 (TE=55 ms, TR=10000 ms). Fifty two ascending slices are acquired with a thickness of 2 mm.

Whole brain 3D high resolution T1-weighted volume (MPRAGE): Acquisition matrix of 256x256 with in-plane resolution 1x1 mm^2 is used for 160 slices of 1 mm thickness in sagittal orientation

Resting State fMRI – 15 minutes eyes closed resting state condition. 430 volumes of standard functional images for each scan (EPI; TR=2100ms, TE=35ms, flip angle 90º; 64x64x35 matrix, resolution 3.25*3.25*3mm).
